# CVD-Synthesis of N-CNT Using Propane and Ammonia

**DOI:** 10.3390/ma15062241

**Published:** 2022-03-18

**Authors:** Valery Skudin, Tatiana Andreeva, Maria Myachina, Natalia Gavrilova

**Affiliations:** 1 Department of Chemical Technology of Carbon Materials, Mendeleev University of Chemical Technology of Russia, Miusskaya Sq., 9, 125047 Moscow, Russia; skudin.v.v@muctr.ru (V.S.); kpolyashova@yandex.ru (T.A.); 2 Department of Colloid Chemistry, Mendeleev University of Chemical Technology of Russia, Miusskaya sq., 9, 125047 Moscow, Russia; gavrilova.n.n@muctr.ru

**Keywords:** N-CNT, CVD-synthesis, carbon nanotube, XPS, TEM, nitrogen doping by ammonia

## Abstract

N-CNT is a promising material for various applications, including catalysis, electronics, etc., whose widespread use is limited by the significant cost of production. CVD-synthesis using a propane–ammonia mixture is one of the cost-effective processes for obtaining carbon nanomaterials. In this work, the CVD-synthesis of N-CNT was conducted in a traditional bed reactor using catalyst: (Al_0,4_Fe_0,48_Co_0,12_)_2_O_3_ + 3% MoO_3_. The synthesized material was characterized by XPS spectroscopy, ASAP, TEM and SEM-microscopy. It is shown that the carbon material contains various morphological structures, including multiwalled carbon nanotubes (MWCNT), bamboo-like structures, spherical and irregular sections. The content of structures (bamboo-like and spherical structure) caused by the incorporation of nitrogen into the carbon nanotube structure depends on the synthesis temperature and the ammonia content in the reaction mixture. The optimal conditions for CVD-synthesis were determined: the temperature range (650–700 °C), the composition (C_3_H_8_/NH_3_ = 50/50%) and flow rate of the ammonia-propane mixture (200 mL/min).

## 1. Introduction

Steady interest in carbon nanotubes (CNTs) is due to their unique properties; already today, they find: practical applications—for the creation of fire-retardant materials, fuel cell electrodes, in catalysis—as catalyst supports, in nanoelectronics—for the creation of one-dimensional conductors, nanosized transistors, supercapacitors, in technology—as additives to polymer and inorganic composites to increase mechanical strength, electrical conductivity and heat resistance [1,2,3,4,5,6,7,8].

A new approach to changing the chemical and electrical properties of CNTs is the modification of the carbon structure by a heteroatom, nitrogen. Currently, to obtain nitrogen-doped carbon nanotubes (N-CNTs), methods and approaches based on the direct formation of material from a nitrogen-containing carbon precursor or on the thermal treatment of undoped CNTs in a nitrogen-containing atmosphere are being developed [9,10,11,12,13,14].

The development of new economical methods for the synthesis of carbon nanomaterials in large quantities is a very urgent task because their widespread use is currently constrained by their high cost, which does not allow the use of N-CNTs on an industrial scale. Admittedly, the most flexible, providing a variety of possible synthesis modes, is the chemical vapor deposition (CVD) method [2].

It is known that the qualitative and quantitative composition of carbon nanotubes obtained by this method depends on the temperature, duration of synthesis, catalyst and gas mixture compositions [15,16,17,18,19,20,21].

It is known that the growth mechanism of N-CNTs is different from the CNT mechanism only by fact that the destruction of nitrogen precursor leads to the formation of nitrogen which diffuses like carbon to the catalyst volume [21].

N-CNT can form bamboo-like or spherical section structures. There are four stages reported in the literature model:

(1) the catalyst reacts with carbon, forming carbide particle;
(2) carbon is forming graphite layer on the surface of carbide particle;
(3) new layers of graphite are formed with a cup-shaped structure;
(4) the cup slides leaving a gap at the tip of the particle [22].

The choice of the catalyst composition for the synthesis of N-CNT by the method of direct incorporation of nitrogen into the carbon structure was made on the basis of preliminary experiments and literature analysis [23,24,25]. The choice of catalyst composition was justified by the following considerations. We proceeded from the fact that the catalyst should have the ability to:

- form metastable carbides;
- accelerate the reactions of dissociation of hydrocarbons with the formation of carbon;
- form metastable nitrides;
- to accelerate the reactions of dissociation of nitrogen-containing compounds, with the formation of nitrogen.

These conditions are satisfied by elements of groups VI and VIII, which can be found in the compositions of catalysts for dehydrogenation, oxidative conversion, and dissociation of hydrocarbons. Most of these catalysts containing these elements simultaneously exhibit catalytic activity in the reaction of synthesis-dissociation of ammonia. The most accessible and cheap, at the same time, are compounds of Fe, Co, Ni, Mo, etc. [1,26].

Compounds of groups III, IV and VI can be considered as promoters that ensure the stability of the catalyst structure under the reaction conditions (Al, Cu, Mo) or as a support (Si, Al). In this case, compounds of VI and VIII groups can participate as a catalyst in both reactions [2].

The choice of the composition of the initial gas mixture is determined by availability and price. It is clear that CH_4_ and NH_3_ are the most accessible for these purposes, but despite this, only a few publications were devoted to the N-CNT synthesis using ammonia and propane [27,28,29]. These compounds are considered among the most stable when heated, but the dissociation reactions of methane and ammonia on iron and cobalt start at very different temperatures. Approximate decomposition temperature ranges for methane: 600–800 °C; propane: 400–700 °C; ammonia: 300–500 °C [29,30]. At high temperatures in the presence of catalysts in a methane-hydrogen mixture, it is difficult to maintain a high concentration of ammonia, since its dissociation begins at a noticeable rate already at temperatures of 300–350 °C. As a result, the concentration of nitrides on the surface of the catalysts and the content of ammonia in the gas mixture at N-CNT synthesis temperatures will be extremely low; this will not allow getting products with a high nitrogen content in them. In this work, these considerations substantiated the choice of propane (C_3_H_8_), which exhibits high reactivity even at a temperature of 600 °C. Therefore, a mixture of propane with ammonia in various ratios was used as the initial mixture in this work. The novelty of this work is due to the possibility of obtaining nitrogen-doped carbon nanomaterials using inexpensive precursors and at a relatively low temperature of 650 °C. In the future, the development of a method for obtaining a material with a certain morphological structure and a certain nitrogen content will make it possible to evaluate the mechanism of the formation of carbon nanotubes through the stage of formation of nitrides.

The aim of this work was to establish the dependence between the nitrogen content in the N-CNT, the composition of the initial mixture of propane and ammonia and synthesis temperature, and also established the morphological composition of the synthesized carbon materials.

## 2. Materials and Methods

### 2.1. Materials

For the synthesis of N-CNTs by chemical vapor deposition, the following gases were used: nitrogen—N_2_ (99.99%), liquefied propane C_3_H_8_ (CH_4_-0.3%, C_2_H_6_-4, 7%, C_3_H_8_-95%), anhydrous liquefied ammonia NH_3_ (99.9%). All gases were purchased by NII KM (Moscow, Russia).

To obtain the catalyst, iron (III) nitrate, Fe(NO_3_)_3_·9H_2_O nonahydrate; cobalt (II) nitrate Co(NO_3_)_2_ 6H_2_O hexahydrate; aluminum nitrate nonahydrate Al(NO_3_)_3_·9H_2_O; aminoacetic acid (glycine) H_2_NCH_2_COOH; ammonium paramolybdate (NH_4_)_6_Mo_7_O_24_ 4H_2_O. All reagents were of the reagent grade and were purchased by CT Lantan (Moscow, Russia).

### 2.2. Synthesis of Catalyst (Al_0,4_Fe_0,48_Co_0,12_)_2_O_3_ + 3% MoO_3_

In a beaker, weighed portions of crystalline hydrates of iron (III) nitrates (2.852 g), cobalt (0.493 g), aluminum (2.119 g) and glycine (1.711 g) are placed. Pure water (2 mL) is added to a sample of crystalline ammonium paramolybdate and heated to 40 °C, stirring until the salt is completely dissolved. Then, the resulting solution of ammonium paramolybdate is transferred into a beaker with weighed portions of salts, the mixture is stirred until complete dissolution. The mixture is heated and stirred until a clear solution of intense red-brown color is formed for 1 h at a temperature of 40 °C. The resulting solution is transferred to a porcelain cup, which is placed in a muffle furnace preheated to 550 °C for 10 min, then removed, cooled to room temperature.

### 2.3. Synthesis of Nitrogen-Doped Carbon Nanotubes

Synthesis of N-CNTs was carried out in a horizontal steel reactor with a fixed catalyst bed with a constant catalyst mass0.05 g (Al_0,4_Fe_0,48_Co_0,12_)_2_O_3_ + 3% MoO_3_). The synthesis was carried out in the temperature range of 650–800 °C for 1 h at a constant total flow rate of 200 mL/min. Samples were obtained at various ratios of propane/ammonia (C_3_H_8_/NH_3_, vol%): 100; 25/75; 50/50; 75/25; 90/10. 

The flow rate of propane and ammonia was set using RRG-12 flow regulators (Eltochpribor, Zelenograd, Russia). The temperature regime for the synthesis of carbon nanotubes was set using a temperature controller TERMODAT-17E6 (PP “Control Systems”, Perm, Russia). The flow rate of the product mixture was determined with an ADM G6691A flow meter (Agilent Tech., Santa-Clara, CA, USA). The ammonia flow rate at the outlet of the reactor was determined by gas titration.

### 2.4. Characterization of Nitrogen-Doped Carbon Nanotubes

The sizes of the carbon nanotubes were determined by LEO 912AM Omega (Carl Zeiss, Oberkochen, Germany) transmission electron microscope. Images were acquired at 100 kV accelerating voltage. The analysis of the microphotographs and the calculation of particle sizes were carried out using the Image Tool V.3.00 (Image Tool Software, UTHSCSA, San Antonio, TX, USA). At least 100 particles per sample were processed. The outer and inner diameters of the nanotubes were determined.

The morphology of carbon materials was studied using a scanning electron microscope (SEM). The micrographs of the samples were taken on JSM 6510 LV + SSD X-MAX microscopes (JEOL, Tokyo, Japan) at an accelerating voltage of 20 kV.

The XPS spectra were recorded using ESCA X-ray photoelectron spectrometer (OMICRON Nanotechnology GmbH, Taunusstein, Germany). The samples of N-CNT investigated by XPS spectroscopy contained catalyst.

The parameters of the porous structure of the samples were calculated based on the isotherm of low-temperature nitrogen adsorption. The studies were carried out on a Gemini VII analyzer (Micromeritics, Norcross, GA, USA) at the Center for Shared Use. DI. Mendeleev. The specific surface area was determined by the BET method. The total pore volume was found from the maximum value of the relative pressure, equal to 0.995. The predominant pore diameter was calculated using the BJH method.

## 3. Results

### 3.1. Influence of Flow Rate and Composition of the Initial Gas Mixture

It is known that ammonia on iron catalysts can dissociate at atmospheric pressure already at temperatures of 300–350 °C. Hydrocarbon gases under these conditions remain practically inert and begin to dissociate at a noticeable rate at temperatures above 600 °C; therefore, it is proposed to use propane as a carbon-containing precursor.

In order to maintain the ammonia concentration under the synthesis conditions at a sufficiently high level, it is necessary to reduce the residence time of the reaction medium in the reactor by increasing its flow rate and lowering the temperature in the reactor.

As can be seen from the results in Table 1, at a temperature in the reactor of 800 °C and a change in the mole flow of pure ammonia at the inlet to the reactor from 18.7 to 37.3 kmole/s, the mole flow of ammonia at the outlet remains practically unchanged. The result obtained indicates that in the studied range of mole flow rates in the outgoing mixture, an equilibrium concentration of unreacted ammonia is established, the level of which is determined by the temperature in the reactor. As the temperature in the reactor decreases, the flow rate and the equilibrium concentration of ammonia under stationary conditions increase.

We concluded that it is not advisable to increase the ammonia concentration at the reactor outlet by reducing the contact time (or, which is also an increase in the linear velocity), since this can lead to catalyst carryover from the reactor and may be accompanied by an unsustainable increase in the consumption of raw materials. Secondly, as can be seen from the table, it is more rational to reduce the temperature in the reactor.

For the synthesis of carbon nanotubes, the value of 26/1 kmol/s (200 mL/min) was chosen as the optimal flow rate of the initial mixture of propane and ammonia, while the synthesis of the carbon material was carried out at different ammonia contents in the initial mixture (C_3_H_8_/NH_3_, vol%): 100; 25:75; 50:50; 75:25; 90:10. The initial temperature for synthesis was chosen as 650 °C.

In order to establish the dependence of the amount of nitrogen introduced into CNTs on the different compositions of the initial gas mixture, as well as to determine the electronic state of atoms on the surface of the material under study, the obtained samples were investigated by X-ray photoelectron spectroscopy.

**Figure 1:**
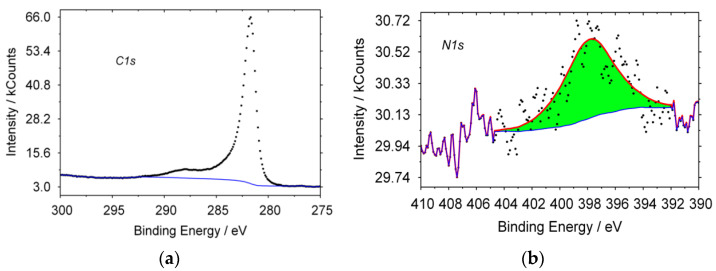
Typical XPS spectra for CNT, synthesized from propane: (**a**) carbon, (**b**) nitrogen.

In Figure 1, the typical XPS spectra of CNT, obtained from propane are shown. For carbon, the line shape of the spectrum has a maximum with E = 284.6 eV, which is typical for sp^2^ hybridization carbon structures. A characteristic peak for nitrogen is observed; in a pyridine-like (398.8 eV) state, other forms of nitrogen are absent. The formation of a product containing nitrogen in its structure could occur only from a mixture of gases that was formed when nitrogen was supplied to the reactor during its heating or cooling. Therefore, N_2_ can also be a nitrogen precursor gas, under the given synthesis conditions. 

**Figure 2:**
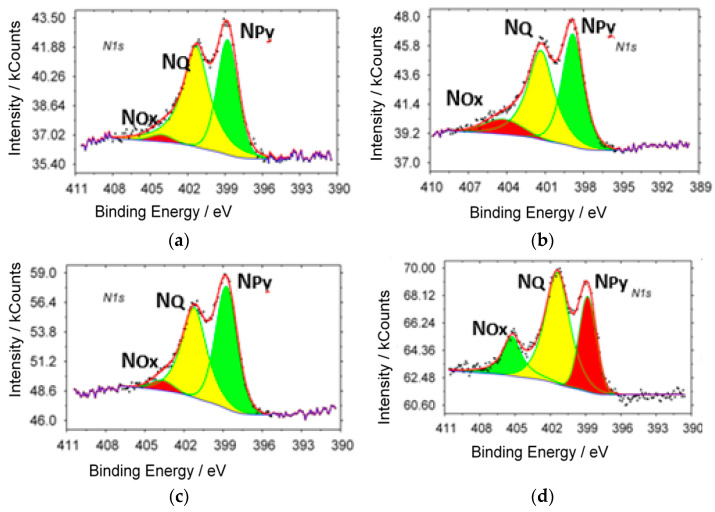
XPS spectra of the samples with different ammonia content in initial mixture: (**a**) 75%; (**b**) 50%; (**c**) 25%;(**d**) 10%.

XPS spectra (N_1s_) of samples with different ammonia content in the initial gas mixture are shown in Figure 2. Characteristic peaks of different state of nitrogen are observed in all investigated samples: in the pyridine-like (N_Py_, 398.8 eV), graphite-like (N_Q_ 401.7 eV) states and oxidized forms of nitrogen (N_Ox_ 405.6 eV).

The content of the different states of nitrogen, carbon and oxygen in synthesized samples are presented in Table 2.

**Figure 3:**
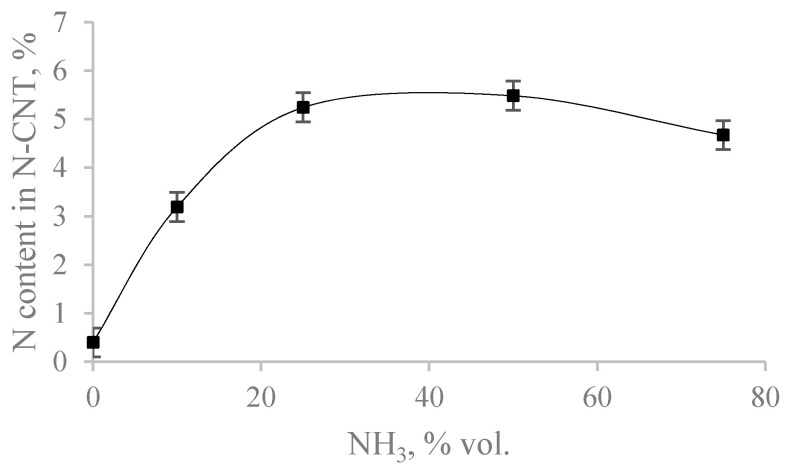
Dependence of N_total_ content in samples on NH_3_ content in the mixture.

**Figure 4:**
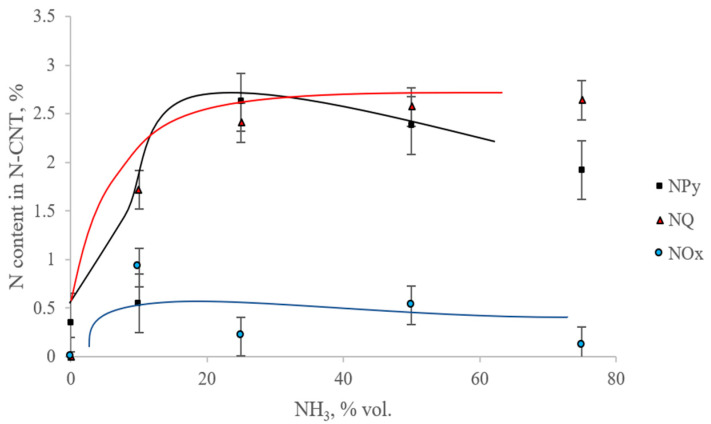
Dependence of the content of various forms of nitrogen on the content of NH_3_ in the mixture.

Figure 3 and Figure 4 show the dependences of the content of total nitrogen and various forms of nitrogen on the initial content of ammonia in the reaction mixture.

From Figure 3 and Figure 4 it can be seen that with an increase in the ammonia content in the initial gas mixture, the total nitrogen content in the samples first increases to the maximum nitrogen content in the sample, synthesized from a mixture of C_3_H_8_/NH_3_ = (50/50%), and then decreases. In this case, the maximum content of the pyridine-like form of nitrogen is observed in the sample, which is synthesized from a mixture of C_3_H_8_/NH_3_ (75/25%). A graphite-like form is seen in the sample C_3_H_8_/NH_3_ (25/75%). The smallest content of oxidized forms of nitrogen contains the N-CNT, synthesized using C_3_H_8_/NH_3_ (25/75%).

According to the literature, the maximum nitrogen content in a doped carbon material can reach about 10%; however, a high nitrogen content is not always justified from the point of view of further use, including as catalyst supports [6,8,31]. Significant incorporation of nitrogen into the structure of carbon nanotubes occurs at a high level of ammonia; however, at a concentration of more than 50%, the process of nitrogen doping slows down, and such an effect of the ammonia content may be due to the different mechanism of formation of nitrogen-doped structures [8]. To establish the mechanism, first of all, it is required to trace which phase transformations the catalyst undergoes. We assume that nitrides or carbonitrides are formed during CVD-synthesis, and the ammonia concentration has a direct effect on this process.

**Figure 5:**
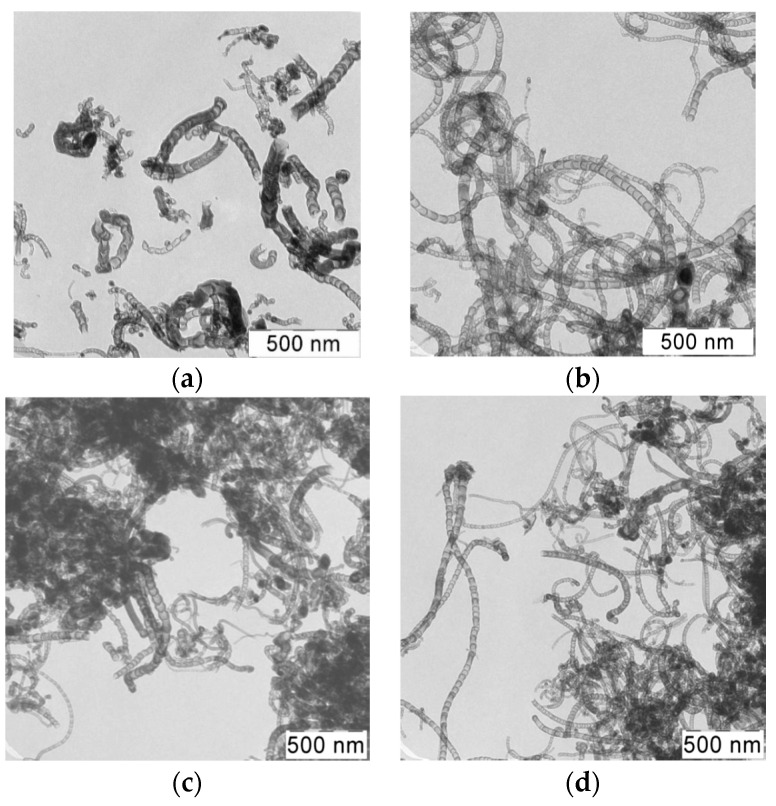
(**a**,**b**) TEM—images of N-CNT obtained using a gas mixture of C_3_H_8_/NH_3_ (50/50%); (**c**) 25/75%; (**d**) 75/25%; at a temperature of 650 °C.

Figure 5 shows TEM-images of samples of synthesized N-CNT using a gas mixture with a different ratio of C_3_H_8_/NH_3_ at a temperature of 650 °C.

**Figure 6:**
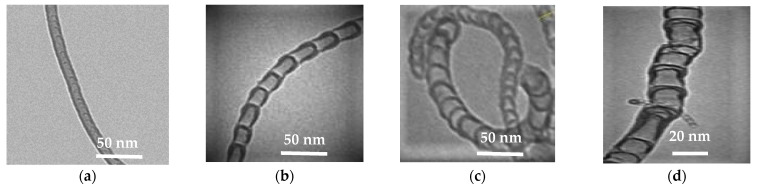
Morphological structures of CNT: (**a**) MWCNT; (**b**) bamboo-like structures; (**c**) spherical sections; (**d**) irregular structures.

As can be seen from the images, the synthesized product is presented by different structures CNTs. There are several types of morphological structures found in the obtained products of nitrogen-doped carbon materials, including multiwalled carbon nanotubes (MWCNT), bamboo-like structures, spherical and irregular sections [13,25]. These types of structures are presented on Figure 6.

From a review of the literature, we can conclude that the nanotubes with bamboo-like, spherical and irregular structures (Figure 6b–d) are most likely nitrogen-doped carbon materials [13,25]. 

Based on the results of transmission electron microscopy of the samples, Table 3 contains the percentage of different type structures in samples, synthesized in the presence of NH_3_ content, and the values of predominant inner and outer diameters. 

It can be seen that the composition of the initial gas mixture affects the morphology, at various ratios of initial gases, certain morphological structures prevail in the product.

With an increase in the content of ammonia in the mixture, there is an increase in the content of bamboo-like structures and a decrease in the content of irregular structures. For the sample synthesized from C_3_H_8_/NH_3_ (50/50%) mixture, the quantitative ratio of all the observed structures occupies an intermediate position with respect to other samples. It also corresponds to the highest total content of fibers with bamboo-like and spherical sections (81.8%).

The values of the predominant outer and inner diameters lie in the range from 31 to 24 and from 24 to 15, respectively. Despite the change in the fractional composition of the carbon material with a change in the initial content of ammonia, no significant difference in the sizes of nanotubes is observed. 

### 3.2. Temperature Effect

The choice of synthesis temperature is based on the dependence of the content of nitrogen atoms embedded in the CNT structure. For this purpose, several CNT samples were synthesized in an ammonium-propane mixture at several temperatures at a constant ratio of ammonia and propane in the initial gas mixture (50/50%). The Table 4 shows the values of product yield and residual catalyst content.

An important parameter in the synthesis of CNTs is their yield, which is determined by the ratio of the mass of the formed product to the mass of the initial catalyst. As can be seen from the presented data, with an increase in the synthesis temperature, an increase in the yield of N-CNTs is observed; moreover, the increase in initial ammonia content leads to the decrease of the N-CNT yield.

It is known that the amount of nitrogen contained in CNTs is affected by the conditions of synthesis [22,31]. Table 5 contains the results of XPS-analysis for samples, synthesized at different temperature and constant gas mixture (50/50%). 

From the data presented in Table 5, it can be seen that with an increase in the process temperature from 650 °C to 800 °C, a general decrease in the nitrogen content in N-CNTs is observed. This result may be due to a decrease in the concentration of ammonia in the gas phase.

It can be also seen that a decrease in temperature promotes the synthesis of materials containing non-oxidized forms of nitrogen incorporated into the carbon structure. An increase in the synthesis temperature leads to the appearance of oxidized forms of nitrogen. At temperatures of 700 °C and 750 °C, nitrogen in the tubes is in two states: pyridine-like and graphite-like, while the content of nitrogen in the graphite-like state is higher than in the pyridine-like state at any synthesis temperature. With an increase in temperature to 800 °C, a third form of nitrogen appears: oxidized. In addition, oxygen was found in the product, the content of which decreases with increasing synthesis temperature. One of the reasons for the appearance of oxygen in the samples, is residual catalyst in N-CNT, which components can be only partially reduced to a metallic state.

**Figure 7:**
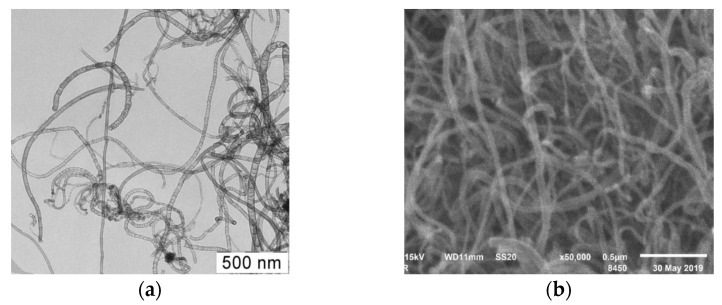
TEM (**a**) and SEM-image (**b**) of N-CNT obtained using a gas mixture of C_3_H_8_-NH_3_ (50/50%) at a temperature of 800 °C.

Figure 7 shows TEM and SEM-images of samples of synthesized N-CNT using a C_3_H_8_/NH_3_ (50/50%) gas mixture at a temperature 800 °C.

As can be seen from the presented figures, the carbon nanomaterial is presented by nanotubes of various morphologies, including multiwalled carbon nanotubes (MWCNT), bamboo-like structures, spherical and irregular sections, similar to the material synthesized at 650 °C.

Based on the obtained TEM-images, the predominant inner and outer diameters and percentage of different morphological structures of nitrogen-doped CNTs were calculated. These results are presented in the Table 6.

It can be seen that the synthesis temperature affects the fractional composition of the resulting carbon material. With an increase in temperature, the content of bamboo-like and irregular structures increases, and the content of structures with spherical sections decreases. Based on the fact that only bamboo-like and spherical structures can be attributed to nitrogen-doped carbon nanotubes, the optimal temperature range for synthesis is 650 °C. 

The values of the predominant outer and inner diameters lie in the range from 26 to 20 and from 16 to 8, respectively. There is no significant change in the particle size with an increase in the synthesis temperature.

**Figure 8:**
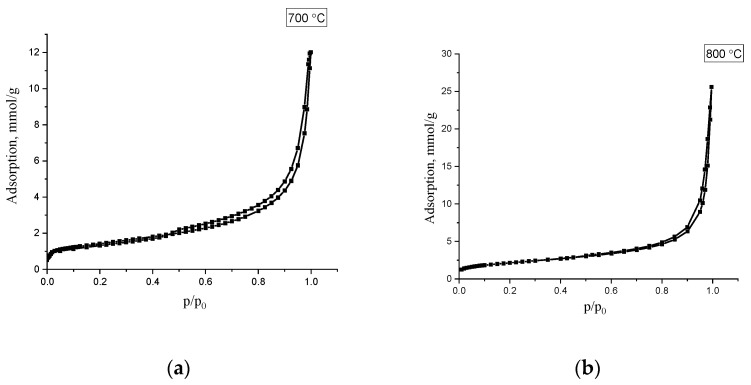
Low-temperature nitrogen adsorption isotherms for N-CNT, synthesized at the temperature of 700 °C (**a**) and 800 °C (**b**).

For investigating of doped N-CNT porous structure samples o with a total nitrogen content (N)_total_ of 4.6 and 2.3%, obtained at temperatures of 700 and 800 °C, were studied using low-temperature nitrogen adsorption, which are presented on the Figure 8.

The isotherms refer to Type II according to the De-Boer classification, and indicates the occurrence of polymolecular adsorption. Based on the obtained isotherms, the main characteristics of the porous structure of were calculated: specific surface area, pore volume, and predominant pore diameter. The obtained values are shown in Table 7.

As can be seen from the presented data, the studied samples of carbon material have mesoporous structure, the specific surface of which is 113 and 215 m^2^/g. The pore volume of the samples is 0.4 and 1.2 cm^3^/g, the main contribution to which is made by mesopores, which are formed due to the interparticle volume between carbon nanotubes. Mesopore size distribution calculated BJH method (Barrett–Joyner–Halenda) shows that the predominant pore size is about 3–4 nm.

An increase in the surface area and pore volume may be associated with an increase in the content of amorphous carbon with an increase in the synthesis temperature.

## 4. Discussion

The main purpose of this work is to choose the conditions for a simple and cost-effective process for obtaining N-CNTs, in which the incorporation of the N atom into the structure of nanotubes will be possible. The carbon material was obtained by embedding a carbon material into the crystal lattice during synthesis using a (Al_0.4_Fe_0.48_Co_0.12_)_2_O_3_ catalyst doped with MoO_3_ (3%) and a propane–ammonia gas mixture. The choice of ammonia and propane as a precursor of the doped carbon material was due to the low cost and the possibility of obtaining the material already at a temperature of 650 °C. An analysis of the literature data showed that this temperature of N-CNT synthesis is relatively low compared to other variants of CVD-synthesis using light hydrocarbons [22].

The content of nitrogen embedded in the crystalline structure of carbon varied due to changes in the ammonia content in the reaction mixture and the synthesis temperature. A total nitrogen content of about 5% has already been achieved with an ammonia content of 25% in the mixture. A further increase in the ammonia content to 90% did not lead to a significant change in the content of total nitrogen; however, the propane/ammonia ratio affects the content of a certain form of nitrogen (graphite-like, pyridine-like and oxidized forms of nitrogen), as well as the fractional composition of N-CNT. According to the TEM results, it was found that the synthesized carbon material contains various morphological structures, including multiwalled carbon nanotubes (MWCNT), bamboo-like structures, spherical and irregular section structure [26]. In accordance with the literature data, only bamboo-like and spherical section structures can be classified as structures in which nitrogen is embedded in the crystalline structure of carbon [13,25]. It was found that the maximum number of such structures is formed when using an equimolar propane/ammonia mixture. 

The next part of the work was devoted to establishing the influence of the synthesis temperature on the N content and the morphological composition of the carbon material. A series of experiments was carried out at a constant propane/ammonia equimolar ratio; it was found that with an increase in temperature from 650 to 800 °C, the product yield increases, but the total nitrogen content decreases from 5.5 to 2.3%. In addition, the analysis of TEM and SEM images showed that there is a change in the content of various morphological structures; there is a decrease in the content of the spherical section structure and irregular structures begin to accumulate. An increase in the product yield, accumulation of irregular structures, and an increase in the specific surface area of the final product indicate the accumulation of amorphous carbon, which in turn leads to a deterioration in the quality of the resulting product. Based on this, it is advisable to carry out the synthesis of N-CNT at temperatures not exceeding 700 °C. 

The temperature dependence of the growth rate of N-CNT can be related to several factors such as concentration, diffusion rate, and growth rate at the interface between the catalyst and the formed nanotube. With an increase in temperature, the rate of diffusion of carbon and nitrogen atoms increases significantly, and the growth rate of N-CNT will correspondingly increase [12].

As a result of this work, the optimal conditions for obtaining doped carbon material (at least 80% N-CNT, total nitrogen content at least 5–6%) from readily available precursors were established. Further studies of CVD-synthesis of N-CNT using propane–ammonia mixture can be directed to: detailed consideration of the growth process of carbon nanotubes and phase transformations of the catalyst during growth; an increase in the activity of catalysts based on Al, Co, Fe oxides and a search for promoters, including molybdenum oxides.

## 5. Conclusions

In this work, nitrogen-doped carbon materials were synthesized using propane and ammonia by the CVD method. It was shown that the total nitrogen content, as well as the content of a certain form of nitrogen (graphite-like, pyridine-like and oxidized forms of nitrogen) depend on the initial ammonia content in the reaction mixture and the synthesis temperature. 

The optimal conditions for N-CNT synthesis were chosen as follows: mixture flow rate, 200 mL/min; the composition of the reaction mixture—C_3_H_8_/NH_3_ (50/50%); temperature range—650–700 °C. Under these conditions, a mesoporous carbon material is formed with a total nitrogen content of about 5% and with a content of nitrogen-doped structures (bamboo-like and spherical) of at least 80%. 

## Figures and Tables

**Table 1:** Dependence of the ammonia flow rate at the outlet of the reactor on its flow rate at the inlet and the temperature in the reactor.

Temperature, °C	Initial Ammonia Mole Flow Rate, kmol/s	Outlet Ammonia Mole Flow Rate, kmol/s
800	18.7	1.4
	22.4	1.4
	26.1	1.4
	29.9	1.4
	37.3	1.5
750	26.1	2.0
700	26.1	3.3
650	26.1	5.1

**Table 2:** Percentage of elements in N-CNT at 650 °C with different ammonia content in initial mixture.

NH_3_ Content, % vol.	-	10	25	50	75
C_1s_, % 284.6 eV	98.5	94.8	88.2	93.4	83.6
(N)_total_, %	0.4	3.2	5.2	5.5	4.7
(N_1s_ 398.8 eV) N_Py_, %	0.4	0.6	2.6	2.4	1.9
(N_1s_ 401.7 eV) N_Q_, %	-	1.7	2.4	2.6	2.6
(N_1s_ 405.6 eV) N_Ox_, %	-	0.9	0.2	0.5	0.1
(O)_total_, %	1.2	1.4	4.4	1.0	1.2

**Table 3:** Percentage of different morphological structures in the samples obtained at a temperature of 650 °C.

NH_3_ Content, % vol.	10	25	50	75
Morphological Structure	Content, % *			
MWCNT	21.1	5.7	9.1	20.8
bamboo-like structure	21.2	11.4	22.7	43.4
spherical section structure	15.2	68.7	59.1	26.4
irregular structure	51.5	14.2	9.1	9.4
Diameter				
Predominant inner, nm	26	31	24	32
Predominant outer, nm	16	24	15	21

* Percentage of particles of a given morphological structure in the sample according to TEM.

**Table 4:** Product yield of the synthesized samples at the different initial content of NH_3_ and different temperature.

Temperature, °C	Product Yield, g_CNT_/g _catalyst_		
	NH_3_ = 25% vol.	NH_3_ = 50% vol.	NH_3_ = 75% vol.
650	3.7	1.6	0.7
700	-	3.4	-
750	-	4.8	-
800	-	20.1	-

**Table 5:** Percentage of elements in N-CNT at different temperature (NH_3_ content in reagent mixture of 50% vol.).

Temperature, °C	650	700	750	800
C_1s_, % 284.6 eV	93.4	92.7	94.3	96.0
(N)_total_, %	5.5	4.6	3.8	2.3
(N_1s_ 398.8 eV) N_Py_, %	2.4	2.3	1.3	0.5
(N_1s_ 401.7 eV) N_Q_, %	2.6	1.3	1.7	1.2
(N_1s_ 405.6 eV) N_Ox_, %	0.5	0.6	0.7	0.7
(O) _total_, %	1.0	2.7	1.9	1.7

**Table 6:** Percentage of different morphological structures in the samples obtained at different temperature (NH_3_ content in reagent mixture—50% vol.).

Temperature, °C	650	700	750	800
Morphological Structures	Content, % *			
MWCNT	9.1	7.3	24.1	6.5
bamboo-like structure	22.7	26.8	31.5	31.4
spherical section structure	59.1	48.8	18.5	39.3
irregular structure	9.1	17.1	25.9	22.8
Diameter				
Predominant inner, nm	26	25	20	25
Predominant outer, nm	16	12	8	15

* Percentage of particles of a given morphological structure in the sample according to TEM.

**Table 7:** Porous structure of the synthesized samples at the initial content of NH_3_ = 50% vol. and different temperature.

Parameter	Temperature, °C	
	700	800
Surface area, m^2^/g	113	215
Pore volume, cm^3^/g	0.4	1.2
Predominant pore diameter, nm	4	3

## Data Availability

The data presented in this study are available on request from the corresponding author.
